# Two Human Cases of *Rickettsia felis* Infection, Thailand

**DOI:** 10.3201/eid2010.140905

**Published:** 2014-10

**Authors:** Sophie Edouard, Saithip Bhengsri, Scott F. Dowell, George Watt, Philippe Parola, Didier Raoult

**Affiliations:** Aix Marseille Université, Marseille, France (S. Edouard, P. Parola, D. Raoult);; Thailand Ministry of Public Health–US Centers for Disease Control and Prevention Collaboration, Nonthaburi, Thailand (S. Bhengsri, G. Watt);; Centers for Disease Control and Prevention, Atlanta, Georgia, USA (S.F. Dowell)

**Keywords:** Rickettsia felis, rickettsia, bacteria, human infections, fever, PCR, Thailand

**To the Editor:** *Rickettsia felis* is an emerging pathogen responsible for fleaborne spotted fever. This new species was first isolated in 2002 from the cat flea, *Ctenocephalides felis,* which was then identified as the main vector of this rickettsia (*1*). *R. felis* has also been described in other flea, tick, chigger, and mite species (*2*) and more recently, in mosquitoes, which are strongly suspected to play a role in transmission of the bacterium (*3**,**4*).

The first evidence of human pathogenicity of *R. felis* was suspected in a patient from Texas, USA, in 1991 and was confirmed by 3 additional case-patients from Mexico in 2000 (*5*). Evidence suggests that this bacterium has a worldwide distribution; ≈100 reports of human cases have been published (*2**,**6*). Moreover, *R. felis* was identified as a common (3%–15%) cause of fever among febrile patients in tropical Africa (*7*). The bacterium has also been described in Asia, but little is known about cases of infection in humans; only 1 human case was described in Thailand in 2003 (*8*).

We enrolled febrile patients (≥7 years of age) who came to 4 community hospitals, 2 in Chiang Rai (northern Thailand) and 2 in Khon Kaen (northeastern Thailand) during 2002–2005. Acute-phase and convalescent-phase (3–5 weeks later) serum samples were obtained from 2,225 patients and tested for *R. felis* by using an indirect immunofluorescence assay (*9*). Seventeen (0.8%) of 2,225 patients showed evidence of seroconversion (IgG titer ≥1:128 or IgM titer ≥1:64 or a ≥4-fold increase in titer).

Specific real-time PCR (qPCR) for *R. felis* was performed with acute-phase serum samples of these patients with primers and probes specific for *orfB* and *vapB1* genes as described (*7*). DNA was extracted by using the Biorobot EZ1 Workstation (QIAGEN, Courtaboeuf, France), and qPCR was performed by using a CFX96 instrument (Bio-Rad, Marne-la-Coquette, France). DNA from *R. felis* strain URRWFXCal^T^ (*1*) was used as a positive control, and sterile water was used a negative control. The qPCR results were positive (cycle threshold ≤35) for the 2 genes for 2 of the 17 patients; the four 150-bp amplicons were sequenced. Sequences of *orfB* (150/150) and *VapB1* (155/155) showed 100% similarity with the sequence from the complete genome of *R. felis* URRWXCAL^T^ (GenBank accession no. CP000053).

Patient 1, a 20-year-old woman, and patient 2, a 45-year-old man, were from Chiang Rai Province. They both had fever, myalgia, arthralgia, headache, abdominal pain, cough, and chest pain. No rashes, eschars, or lymphadenopathies were noted. In addition, patient 2 had photophobia, had vomited, and reported contact with cats. Both patients reported having contact with others animals and being bitten by insects, including mosquitoes (Table).

**Table:** Characteristics of 2 febrile patients with confirmed *Rickettsia felis* infection, Chiang Rai Province, Thailand*

Characteristics	Patient 1	Patient 2
Age, y	20	45
Sex	F	M
Acute-phase IgG/IgM titer	0/0	0/0
Convalescent-phase IgG/IgM titer	128/32	128/0
Hospitalization	Yes	Yes
Animal exposure within past 2 wk before illness		
Cats	No	Yes
Dogs	Yes	Yes
Rodents	No	No
Insect bites	Yes	Yes
Mosquito bites	Yes	Yes
Laboratory values		
Leukocytes/mm^3^	6,300	4,100
Hemoglobin, g/dL	11.7	16.0
Platelets/mm^3^	103,000	216,000
BUN, mg/dL	62	31.1
Creatinine, mg/dL	3.56	1.1
ALT, IU/dL	44.6	29.1
AST, IU/dL	49.8	30.2
Bilirubin, mg/dL	0.5	0.97
Alkaline phosphatase, IU/dL	132	137

*BUN, blood urea nitrogen; ALT, alanine aminotransferase; AST, aspartate aminotransferase.

*R. felis* DNA was detected in serum samples from these 2 patients with acute febrile illness in Thailand. The immunofluorescent assay, the reference serologic method for diagnosis of infection with *Rickettsia* spp., is known to show cross-reactivity with other *Rickettsia* spp. Therefore, diagnosis of rickettsial infection should be confirmed by Western blotting or molecular testing. Real-time PCRs are increasingly being used for diagnosis of rickettsioses, including those with *R. felis*, and for vector and reservoir identification (*2*).

The predominant rickettsioses reported in Asia are murine typhus and scrub typhus, which are caused by *R. typhi* and *Orientia tsutsugamushi*, respectively (*8*). To the best of our knowledge, only 12 human cases of *R. felis* infection have been reported in Asia: 3 in Thailand (including these cases), 3 in Sri Lanka, 1 in Laos, 1 in Israel, 1 in Taiwan, and 3 in South Korea (*2**,**8**–**10*). The prevalence of *R. felis* in fleas has been well studied in >20 countries, including Japan, Thailand, Indonesia, Laos, Taiwan, Israel, Afghanistan, and Lebanon (*2*). This bacterium has also been described in mites in Taiwan and South Korea, in chiggers in South Korea, and in ticks in Japan (*2**,**9**,**10*).

The clinical signs and symptoms of *R. felis* infection are now better understood. The more frequent clinical findings reported are nonspecific and include fever, asthenia, headache, maculopapular rash, and inoculation eschar. Neurologic, digestive, and respiratory symptoms are not commonly reported (*2*). These infections could be confused with other rickettsioses or other febrile illnesses, such as malaria. In most regions, laboratory tests are unavailable; consequently, *R. felis* infections are largely underdiagnosed.

The findings of this study indicate that *R. felis* infections may be among the causes of febrile illness in Thailand and highlight the need for physicians to consider this pathogen in the differential diagnosis of diseases in tropical countries and in travelers. Further studies are needed to ascertain risk factors and confirm the causal association and pathology of fleaborne spotted fever in Asia.
